# Coalescent: an open-science framework for importance sampling in coalescent theory

**DOI:** 10.7717/peerj.1203

**Published:** 2015-08-18

**Authors:** Susanta Tewari, John L. Spouge

**Affiliations:** National Center of Biotechnology Information, Bethesda, MD, United States

**Keywords:** Framework, Population genetics, Open-science, Importance sampling, Likelihood, Infinite sites model, Coalescent theory

## Abstract

**Background.** In coalescent theory, computer programs often use importance sampling to calculate likelihoods and other statistical quantities. An importance sampling scheme can exploit human intuition to improve statistical efficiency of computations, but unfortunately, in the absence of general computer frameworks on importance sampling, researchers often struggle to translate new sampling schemes computationally or benchmark against different schemes, in a manner that is reliable and maintainable. Moreover, most studies use computer programs lacking a convenient user interface or the flexibility to meet the current demands of open science. In particular, current computer frameworks can only evaluate the efficiency of a single importance sampling scheme or compare the efficiencies of different schemes in an ad hoc manner.

**Results.** We have designed a general framework (http://coalescent.sourceforge.net; language: Java; License: GPLv3) for importance sampling that computes likelihoods under the standard neutral coalescent model of a single, well-mixed population of constant size over time following infinite sites model of mutation. The framework models the necessary core concepts, comes integrated with several data sets of varying size, implements the standard competing proposals, and integrates tightly with our previous framework for calculating exact probabilities. For a given dataset, it computes the likelihood and provides the maximum likelihood estimate of the mutation parameter. Well-known benchmarks in the coalescent literature validate the accuracy of the framework. The framework provides an intuitive user interface with minimal clutter. For performance, the framework switches automatically to modern multicore hardware, if available. It runs on three major platforms (Windows, Mac and Linux). Extensive tests and coverage make the framework reliable and maintainable.

**Conclusions.** In coalescent theory, many studies of computational efficiency consider only effective sample size. Here, we evaluate proposals in the coalescent literature, to discover that the order of efficiency among the three importance sampling schemes changes when one considers running time as well as effective sample size. We also describe a computational technique called “just-in-time delegation” available to improve the trade-off between running time and precision by constructing improved importance sampling schemes from existing ones. Thus, our systems approach is a potential solution to the “2^8^ programs problem” highlighted by Felsenstein, because it provides the flexibility to include or exclude various features of similar coalescent models or importance sampling schemes.

## Introduction

Felsenstein et al. (1999; Section 14) describes “the 2^8^ programs problem” obstructing computational inference in population genetics, namely, that each variation in a statistical model or computational method requires a new computer program, even if underlying concepts remain similar. For example, importance sampling for population genetic models is an active area of research, but the 2^8^ programs problem obstructs the comparision of novel and existing ideas, because no available computational framework can readily compare different importance sampling proposals. A computer program directly modelling the underlying concepts would provide a systematic solution, but for various reasons, existing implementations do not typically reflect the systematic approach (Stodden, 2013a) characterizing the development of theory. A systematic approach has the potential to improve the reliability of the implementation of base concepts and dramatically reduce the programming effort required to benchmark new ideas.

We therefore took a systems approach to population genetic models and developed a computational framework for importance sampling. Currently, the framework implements a standard neutral coalescent model of a single, well-mixed population of constant size over time under the infinite sites model of mutation. By design, however, it circumvents the 2^8^ programs problem, so that the programming effort to augment a model with a new feature is linear in time (by definitions given by Felsenstein et al. (1999) and its discussion of the 2^8^ programs problem). The framework can compare any subset of proposals programmed into it. In particular, we implemented the three standard proposals (Ethier–Griffiths–Tavaré, Stephens–Donnelly, and Hobolth–Uyenoyama–Wiuf) to compare them within a single framework. Because the framework already implements concepts like “likelihood”, “genealogy”, etc., adding a new proposal only requires specifying the corresponding algorithm.

The systems approach here mirrors our previous approach to computing exact coalescent probabilities (Tewari & Spouge, 2012). Previously, we implemented the computation of the exact likelihood for a general class of coalescent models based on recursions. The general implemention of recursions permits many useful computations (such as counting the total number of ancestral configurations, total number of genealogies etc.) with programming effort only linear in time, as explained above. The implementation permitted us to program and study several competing algorithms for computing exact probabilities (e.g., the forward algorithm of Wu, 2009), and we exemplified its use with both the infinite alleles and infinite sites model of mutation. Although exact probabilities help to benchmark computations and approximations in small datasets, they also aid intuition, with the potential to improve proposals in importance sampling. Thus, our two frameworks have complementary purposes.

## Background

### Infinite-sites model (K69)

Excellent overviews of various coalescent models are available (e.g., Hein, Schierup & Wiuf, 2005; Wakeley, 2009). Here for the sake of completeness, we briefly describe the infinite-sites model (denoted “*K69”,* after Kimura, 1969; also see Watterson, 1975). Most of our notation follows Wakeley (2009).

Consider an aligned sample of DNA sequences, noting that alignment columns can contain gaps. An alignment column lacking gaps is called a “site”, and Model K69 considers only sites. Under Model K69, the sample evolves from most recent common ancestor (MRCA) by reproduction, with occasional mutation at the sites. Model K69 is most suitable for long DNA sequences with low mutation rates, because it permits at most one mutation at each site during the evolution of the sampled sequences. The state of each site in a sampled sequence (its “character”) can therefore be summarized by a binary digit: 0, if the corresponding DNA letter agrees with the MRCA; and 1, otherwise. A site is segregating if some sequences in the relevant sample contain the character 1. Thus, the segregating sites comprise the essential data in the sample. The sample data can be represented as $D=\left[X,\nu \right]$, where *X* is a binary matrix (i.e., ${X}_{i,j}\in \left\{0,1\right\}$) with distinct rows *X_i_* (“haplotypes”) and *ν* is an column vector such that *ν_i_* counts the multiplicity of the haplotype *X_i_* among the sampled sequences. Let *n* denote the total number of alleles i.e., $n=\sum _{\left(i\right)}{\nu }_{i}$. Thus, the character of haplotype *X_i_* at site *j* is ${X}_{i,j}\in \left\{0,1\right\}$. See Table 1 for a sample dataset $D=\left[X,\nu \right]$ similar to Figure 8.6 in Wakeley (2009).

**Figure 1:**
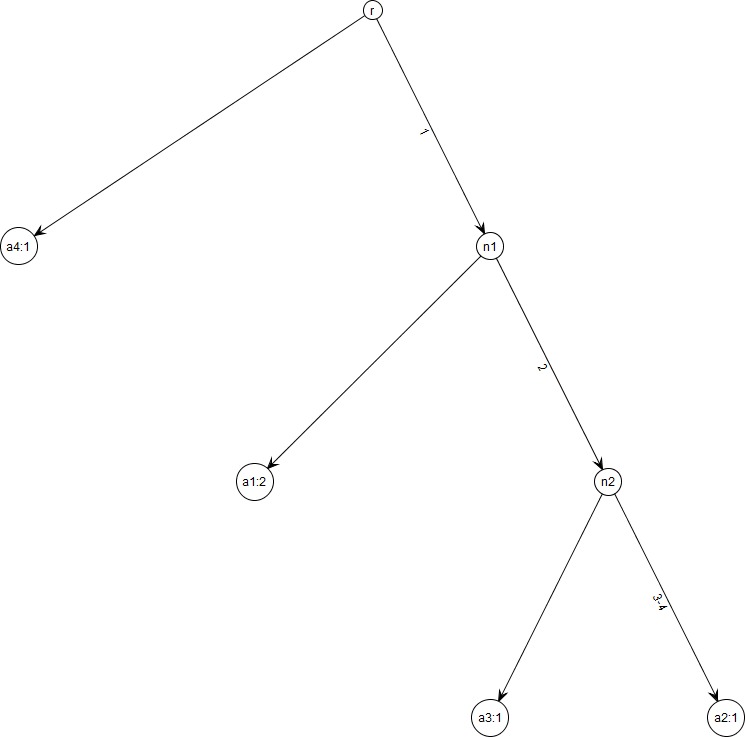
Gene tree for data in Table 1. Note that gene trees are not coalescent trees where time information is present. The leaf nodes correspond to the alleles and their count, e.g., node *a*_1_: *2* indicates allele *a*_1_ with count *2*. Mutations are labelled on the edges and all the children node inherit those mutations, e.g., Mutation *1* is inherited by all the leaf nodes except *a*_4_:*1*. This figure was drawn using the framework in Tewari & Spouge (2012).

As an aid to visualization, data conforming to Model K69 always have a unique *gene tree*. Figure 1, e.g., shows the gene tree corresponding to Table 1. Within a gene tree, the order of mutations on any edge is arbitrary, and permutation of the column order in the haplotype matrix *X* does not affect the gene tree for $D=\left(X,\nu \right)$. Gusfield (1991) gives an efficient algorithm for constructing gene trees.

**Table 1:** Data set for Model K69, similar to Fig. 8.6 in Wakeley (2009). The characters are encoded as 0 and 1. Mutations are encoded as numbers from 1 to 4. The blue cells are the haplotype matrix, whose rows give the characters in each haplotype. The column vector to the right counts each haplotype in the sample data set, so the total number of genetic samples is 5.

Allele	Segregating sites				Count
	*1*	*2*	*3*	*4*	
*a1*	1	0	0	0	2
*a2*	1	1	1	1	1
*a3*	1	1	0	0	1
*a4*	0	0	0	0	1

**Figure 2:**
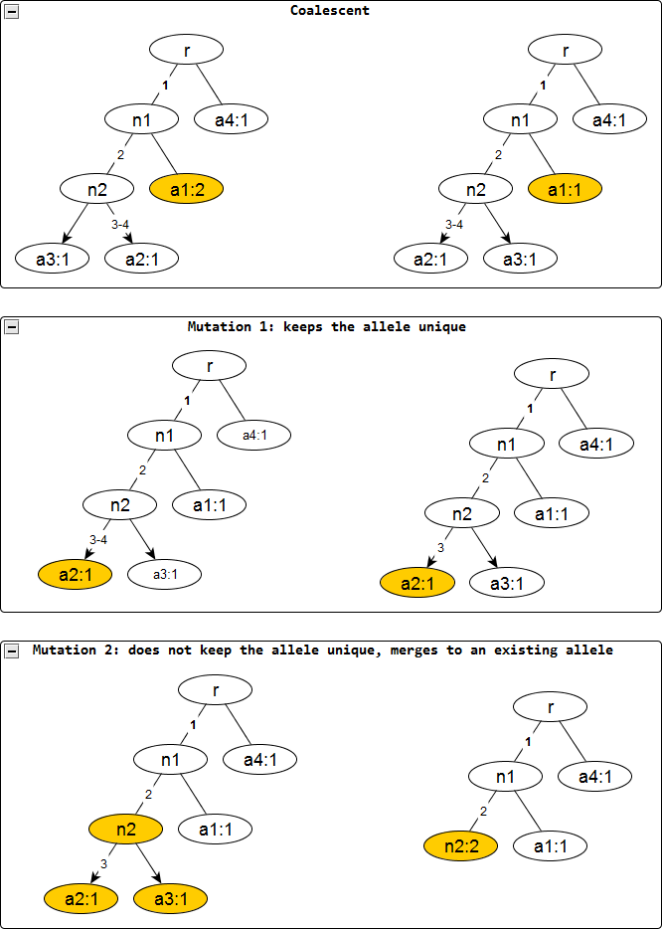
Evolutionary operations in the infinite sites model. The before and after configurations are drawn for each evolutionary operation. The affected nodes are represented in color. Coalescent events reduce the node frequency by *1* but does not affect the edge labels. Mutation events remove the edge labels one at a time. The first kind keeps the affected allele unique whereas the second one does not and merges with an existing allele, incresing the frequnecy of the merged allele by one.

Under Model K69, the MRCA (represented by a matrix with a single row of 0s) evolves into the sample data $D=\left[X,\nu \right]$ by passing stepwise through a sequence of ancestral configurations, which have a form $C=\left[X,\nu \right]$ similar to the sample data. In the following, a “singleton row *i*” is a row with count *ν_i_* = 1. Starting from sample back in time to the MRCA, each step corresponds to one of three possible evolutionary operations on the current ancestral configuration: (1) coalescence (deleting one from a set of identical rows); (2) removing a “type I” mutation, which leaves the sequence still unique (changing the only 1 in some column *j* into 0 and leaves the corresponding singleton row *i* unique in the ancestral configuration); and (3) removing a “type II” mutation, which makes the sequence identical to one or more others (changing the only 1 in some column *j* into 0, to make the corresponding singleton row *i* the same as some other row(s) in the ancestral configuration). Removal in both mutational types I and II is restricted to “the only 1 in some column” and “a singleton row”, because Model K69 permits at most one mutation at each site. Once a mutation is removed, the corresponding site is no longer a segregating site (i.e., the corresponding column in the new binary matrix has only 0s). Figure 2 illustrates these operations. Thus, a computer can efficiently represent the removal of a mutation simply by removing the corresponding column from *X*, a representation we now use. Under the representation, the MRCA becomes an empty matrix with count 1.

To represent the three evolutionary operations mathematically, consider an ancestral configuration $C=\left[X,\nu \right]$, let *e_i_* denote a column vector with 1 in the *i*-th position, and 0 elsewhere. Given *C*, let *A* denote the set of singleton rows *i*, and $A\left(i\right)$ denote column *j* with the smallest index in row *i*, so that row *i* and column $A\left(i\right)$ satisfy the restrictions on mutations of type I (In the following, the arbitrary choice of the smallest column index $A\left(i\right)$ is feasible, because column order is irrelevant to the gene tree.). Let *δ_i_* be the corresponding evolutionary operator that deletes column $A\left(i\right)$ from *X*, creating the new ancestral configuration $\left[{\delta }_{i}X,\nu \right]$. Similarly, let *B* denote the set of singleton rows *i*, each with a single column *j* satisfying the restrictions on mutations of type II. For each *i*, let $B\left(i\right)$ be the row index of the “merge haplotype”, the haplotype that row *i* becomes when the 1 in column *j* is changed to 0. Let Δ_*i*_ be the corresponding evolutionary operator, which deletes row *i* and column *j* from *X*, creating the haplotype matrix Δ_*i*_*X*, and which also deletes the *i*-th row of the column vector *ν*, so the new ancestral configuration is $\left[{\Delta }_{i}X,{\Delta }_{i}\left(\nu +{e}_{B\left(i\right)}\right)\right]$.

Having defined the sample space of ancestral configurations $\left[X,\nu \right]$ and the steps that Model K69 permits, we now determine the corresponding probability measure $p\left[X,\nu \right]$, which implicitly depends on a population mutation parameter *θ*. The MRCA probability is $p\left(\left[\right],\text{\hspace{0.167em} }\left[1\right]\right)\text{\hspace{0.167em}}=\text{\hspace{0.167em}}1.0$, and the probabilities $p\left[X,\nu \right]$ satisfy the recursion

(1) \begin{eqnarray*} n\left(n-1+\theta \right)p\left[X,\nu \right]=\sum _{i:\nu _{i}\geq 2}{\nu }_{i}\left({\nu }_{i}-1\right)p\left[X,\nu -{e}_{i}\right]+\theta \sum _{i:i\in A}p\left[{\delta }_{i}X,\nu \right]+~\theta \sum _{i:i\in B}\left({\nu }_{B\left(i\right)}+1\right)p\left[{\Delta }_{i}X,{\Delta }_{i}\left(\nu +{e}_{B\left(i\right)}\right)\right], \end{eqnarray*}

For introductory examples see Hein, Schierup & Wiuf, 2005; Wakeley, 2009.

### Importance sampling for computing likelihood

Wakeley (2009) gives an overview of importance sampling for computing likelihood in coalescent theory. Briefly, here are the key concepts. To make the dependence of the probability on the mutational parameter *θ* explicit, let $p\left(D;\theta \right)=p\left[X,\nu \right]$ for $D=\left(X,\nu \right)$. The probability of the data in (1) can also be written as the following:

(2) \begin{eqnarray*} p\left(D;\theta \right)=\sum _{G}p\left(D\mid G;\theta \right)p\left(G\right) \end{eqnarray*}

where the sum is over all genealogies *G* consistent with the data *D*. Let $q\left(.\right)$ be any probability measure, and ${E}_{q}\left[.\right]$ be an expectation under $q\left(.\right)$, and define the ratio $w\left(G\right)=p\left(G\right)/q\left(G\right)$. If $p\left(.\right)$ is absolutely continuous with respect to $q\left(.\right)$, i.e., if *q*(*G*) > 0 wherever *p*(*G*) > 0, then

(3) \begin{eqnarray*} p\left(D;\theta \right)=\sum _{G}p\left(D\mid G;\theta \right)\frac{p\left(G\right)}{q\left(G\right)}q\left(G\right)={E}_{q}\left[p\left(D\mid G;\theta \right)\frac{p\left(G\right)}{q\left(G\right)}\right]={E}_{q}\left[p\left(D\mid G;\theta \right)w\left(G\right)\right]. \end{eqnarray*}

Usually, in the context of importance sampling, $p\left(.\right)$ is called the target distribution; $q\left(.\right)$, the trial distribution; and $w\left(.\right)$, the importance sampling weight (Hammersley & Handscomb, 1964; Liu, 2002). Given *R* realizations *G_r_* (*r* = 1, …, *R*) of the genealogy *G* *independently* sampled from the trial distribution $q\left(.\right)$, then the strong law of large numbers implies that with probability 1,

(4) \begin{eqnarray*} p\left(D;\theta \right)=\lim _{R\rightarrow \infty }{R}^{-1}\sum _{r=1}^{R}p\left(D\mid {G}_{r};\theta \right)w\left({G}_{r}\right). \end{eqnarray*}

Thus, importance sampling provides an estimator for the likelihood

(5) \begin{eqnarray*} {\hat {p}}_{I S}\left(D;\theta \right)\approx {R}^{-1}\sum _{r=1}^{R}p\left(D\mid {G}_{r};\theta \right)w\left({G}_{r}\right). \end{eqnarray*}

Equation (1) provides a sequence of steps from a population sample to its most recent common ancestor (MRCA), each step corresponding to a single ancestral coalescence or the loss of a single mutation. A Monte Carlo simulation can therefore assign trial probabilities $q\left(.\right)$ to these time-steps, to create a sequential importance-sampling scheme (Liu, 2002). Many coalescent processes are Markovian, so sequential importance sampling (SIS) is a natural choice to simulate them, because events occurring at different time-steps are independent. The coalescence literature often uses the terminology “proposals” for the sampling choices, although “proposals” does not follow the standard usage in the Monte Carlo literature (The non-standard usage might be derived from the Metropolis method, which accepts or rejects “proposals”). In any case, this paper adheres to standard Monte Carlo terminology (Liu, 2002; Robert & Casella, 2004) whenever there is no conflict with terminology in the coalescent literature.

### Standard sequential samplers

Sequential samplers choose among the evolutionary operations corresponding to the different terms in Eq. (1). Because each operation is determined once the corresponding haplotype *X_i_* in the ancestral configuration $C=\left(X,\nu \right)$ is known, we let $q\left(i\mid C\right)$ with various subscripts denote corresponding trial probability.

#### The Ethier–Griffiths–Tavare (EGT) sequential sampler

The EGT recursion in Eq. (1) directly suggests a sequential sampling scheme (Griffiths & Tavare, 1994):

(6) \begin{eqnarray*} {q}_{G T}\left(i\mid C\right)\propto \left\{\begin{array}{cc} \displaystyle {\nu }_{i}-1&\displaystyle {\nu }_{i}\geq 2\\ \displaystyle \frac{\theta }{n}&\displaystyle i\in A\\ \displaystyle \frac{\theta \left({\nu }_{B\left(i\right)}+1\right)}{n}&\displaystyle i\in B\\ \displaystyle 0&\displaystyle \text{otherwise} \end{array}\right\}. \end{eqnarray*}

#### The Stephens–Donnelly (SD) sequential sampler

Stephens & Donnelly (2000) developed a sampling scheme by characterizing the target distribution and then approximating it with

(7) \begin{eqnarray*} {q}_{\mathit{SD}}\left(i\mid C\right)\propto \left\{\begin{array}{cc} \displaystyle {\nu }_{i}&\displaystyle {\nu }_{i}\geq 2~\text{or}~i\in A~\text{or}~i\in B\\ \displaystyle 0&\displaystyle \text{otherwise} \end{array}\right\}. \end{eqnarray*}

#### The Hobolth–Uyenoyama–Wiuf (HUW) sequential sampler

Hobolth, Uyenoyama & Wiuf (2008) approximated the effects of all mutations on the probabilities for the next step from the sample to the MRCA, to derive

(8) \begin{eqnarray*} {q}_{H U W}\left(i\mid C\right)\propto \left\{\begin{array}{cc} \displaystyle \sum _{m}{u}_{i,m}\left({\theta }_{0}\right)&\displaystyle {\nu }_{i}\geq 2~\text{or}~i\in A~\text{or}~i\in B\\ \displaystyle 0&\displaystyle \text{otherwise} \end{array}\right\}, \end{eqnarray*}

where *θ*_0_ is a fixed value of *θ*,

\begin{eqnarray*} {u}_{i,m}(\theta )=\left\{\begin{array}{cc} \displaystyle {p}_{\theta }\left({d}_{m}\right)\frac{{\nu }_{i}}{{d}_{m}}&\displaystyle {X}_{i,m}=1\\ \displaystyle \left[1-{p}_{\theta }\left({d}_{m}\right)\right]\frac{{\nu }_{i}}{\left(n-{d}_{m}\right)}&\displaystyle {X}_{i,m}=0 \end{array}\right\} \end{eqnarray*}

\begin{eqnarray*} {d}_{m}=\sum _{m}{X}_{i,m}{\nu }_{i} \end{eqnarray*}

and

\begin{eqnarray*} {p}_{\theta }\left({d}_{m}\right)={\frac{\sum _{k=2}^{n-{d}_{m}+1}\frac{{d}_{m}-1}{n-k}\frac{1}{k-1+\theta }\left(\begin{array}{cc} \displaystyle n-{d}_{m}-1\\ \displaystyle k-2 \end{array}\right)\left(\begin{array}{cc} \displaystyle n-1\\ \displaystyle k-1 \end{array}\right)}{\sum _{{k}_{0}=2}^{n-{d}_{m}+1}\frac{1}{{k}_{0}-1+\theta }\left(\begin{array}{cc} \displaystyle n-{d}_{m}-1\\ \displaystyle {k}_{0}-2 \end{array}\right)\left(\begin{array}{cc} \displaystyle n-1\\ \displaystyle {k}_{0}-1 \end{array}\right)}}^{-1} \end{eqnarray*}

\begin{eqnarray*} {p}_{\theta }\left(1\right)=\frac{\frac{1}{n-1+\theta }}{\sum _{{k}_{0}=2}^{n}\frac{1}{{k}_{0}-1+\theta }\frac{{k}_{0}-1}{n-1}}, \end{eqnarray*}

where ${d}_{m}=\sum _{i}{X}_{i m}{\nu }_{i}$ is the total number of alleles containing mutation *m* and ${p}_{\theta }\left({d}_{m}\right)$ is the probability that the next evolutionary operation (coalescence or mutation) involves a row *X_i_* where *X*_*i*,*m*_ = 1 (i.e., haplotype *i* bears mutation *m*) and *u*_*i*,*m*_ denotes the probability of involving row *X_i_* in the next mutation event *m*. The proposal probability in Eq. (8) sums *u*_*i*,*m*_ over all mutations *m* for row *X_i_*.

## Implementation

We now describe the architecture of the framework, diagramming the key classes and interfaces with the unified modelling language (UML), while displaying the various connections and assumptions. The diagrams use standard UML notations (explained in the UML-Wiki). The framework consists of several packages, which progressively narrow the most general concepts down to the specifics of *K69*, the infinite-sites model of mutation.

### Core framework

**Figure 3:**
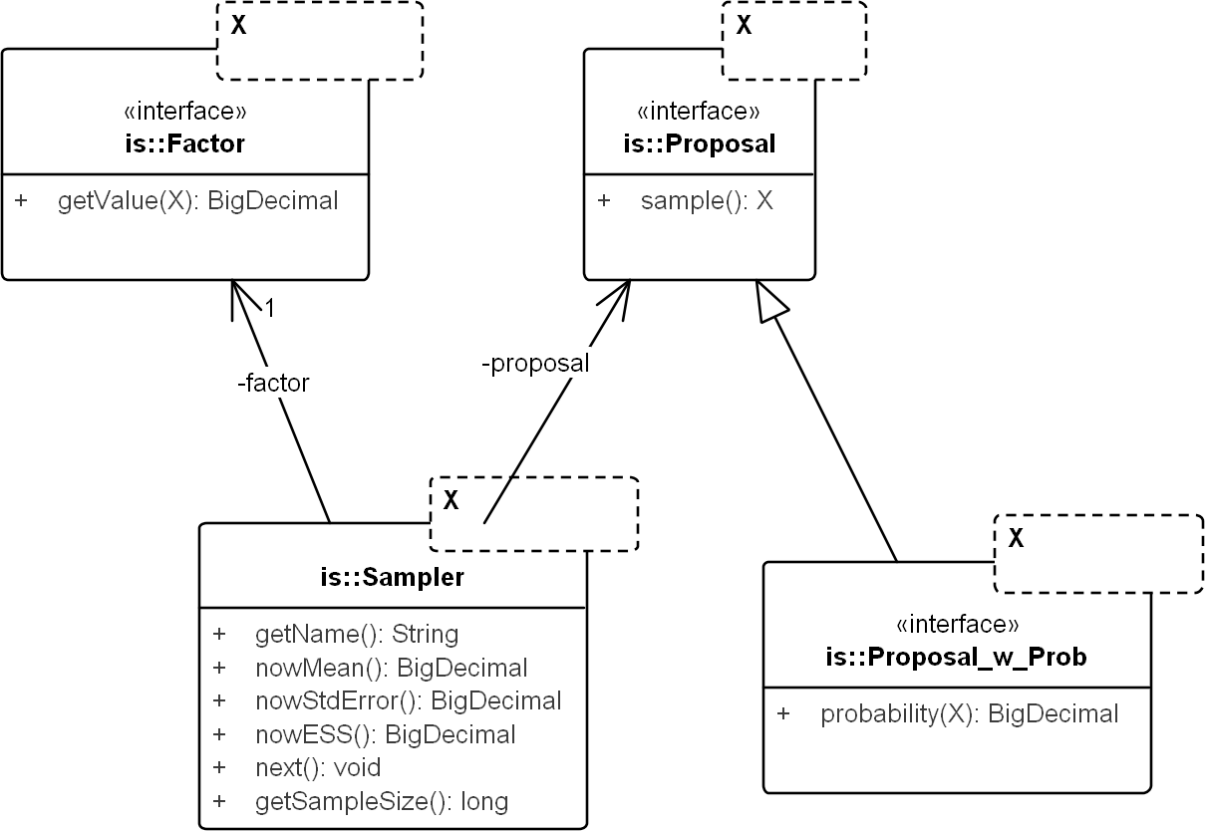
UML diagram of the core importance sampling (IS) framework. The diagram shows key classes corresponding to the concepts of sampler, proposal and importance weight (*Factor*). They are parametric over the domain of sampling, denoted by *X*. For coalescent models, the domain of sampling is *Genealogy*. *Proposal* specifies the sampling algorithm and its extension, *Proposal_w_Prob* specifies its probability distribution. The sampler performs the sampling by repeatedly calling *next()*. The various *nowX(.)* methods give estimates based on the sampled values so far. In text, a sampler is referred by its proposal name when the context is clear.

The core framework models the concepts for any domain of sampling. It corresponds to the package *commons.is*. Figure 3 displays the key classes: *Sampler*, *Proposal*, and *Factor*.

#### Sampler

*Sampler* generalizes Eq. (5) as

(9) \begin{eqnarray*} {\hat {E}}_{q}[Y]=\sum _{r=1}^{R}h\left({Y}_{r}\right)w\left({Y}_{r}\right), \end{eqnarray*}

where $h\left(Y\right)$ is called a “mean function” i.e., a function whose mean (or expectation) is to be computed, and $w\left({Y}_{r}\right)=p\left({Y}_{r}\right)/q\left({Y}_{r}\right)$, $p\left(.\right)$ and $q\left(.\right)$ being the target and trial distributions, respectively. For coalescent models, *Y* corresponds to the genealogy *G*, and $h\left(Y\right)$ denotes the conditional probability of observing data *D* given the genealogy *Y*. Note that for coalescent models, *Y* represents the relevant events in the entire genealogical history of the sample, including coalescent events. To estimate the probabilities in the standard sequential sampling schemes above, take $h\left(Y\right)=1$ identically for all *Y*.

#### Proposal & factor

*Proposal* draws an independent sample *Y* each time *sample()* is called and *Factor* computes $w\left(Y\right)$ in Eq. (9). *Factor* can be created by computing the weight $w\left(Y\right)$ directly (by implementing sub-interface *Proposal_w_Prob*) or by implementing an analytical expression for the ratio, if available (e.g., the so-called “functional path” *F_j_* in Eq. 12 of Griffiths & Tavare, 1994).

### Coalescent models

**Figure 4:**
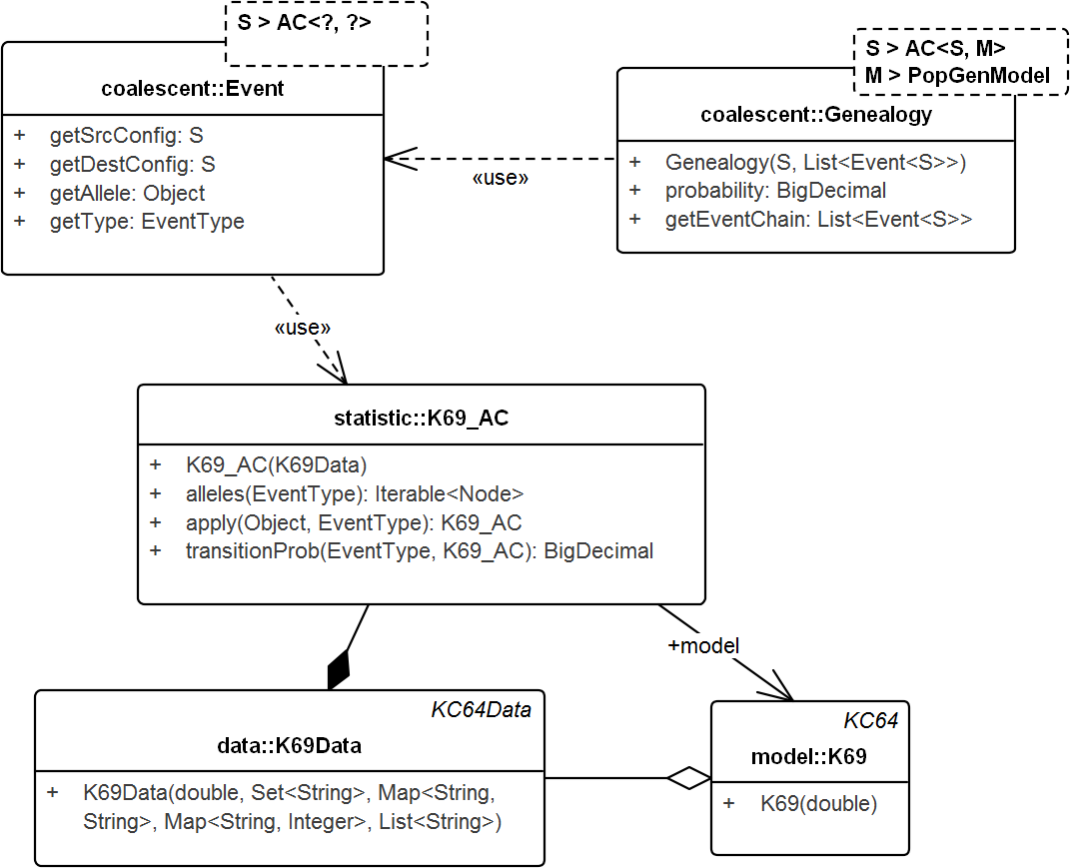
UML diagram of the key classes in Tewari & Spouge (2012) used for sampling genealogies. The diagram shows the connection between *K69_AC* (an *AC* in model *K69*) and *Genealogy*. *AC*s generate Events via method *apply()* recursively and the chain of events in turn create the *Genealogy*. Events do not carry the time information. Its allele property specifies the allele of the source configuration that generated the destination configration. Note that, *Genealogy* is connected to the underlying data and model and carries enough information to compute its own probability. *Event* and *Genealogy* are both generic and work across a range of types. *Genealogy*, for example, would work with any type *S* that inherits from *AC* (such as *K69_AC*) and type *M* that inherits from *PopGenModel* (such as *K69*).

The following subsection describes SIS schemes for coalescent genealogies. Our framework for exact probabilities in coalescent models (Tewari & Spouge, 2012) already contains the general concepts for implementing sequential schemes for specific models. Figure 4 sketches the interactions among these general concepts.

#### Genealogy & AC

*Genealogy* defines the chain of events from the sample to the MRCA. *AC* denotes the ancestral configuration in a generic coalescent model and given the allele and event types, specifies the recursion. Ancestral configuration (K69_AC—for model K69) provides alleles on which events (coalescent, mutation) occur and also generates the resulting ancestral configurations. The recursive events create genealogies that start from the sample and end at the MRCA. The Event object captures the source and destination configurations, the event type, the allele on the source configuration that created the destination configuration, but not the time information.

#### GProposal

**Figure 5:**
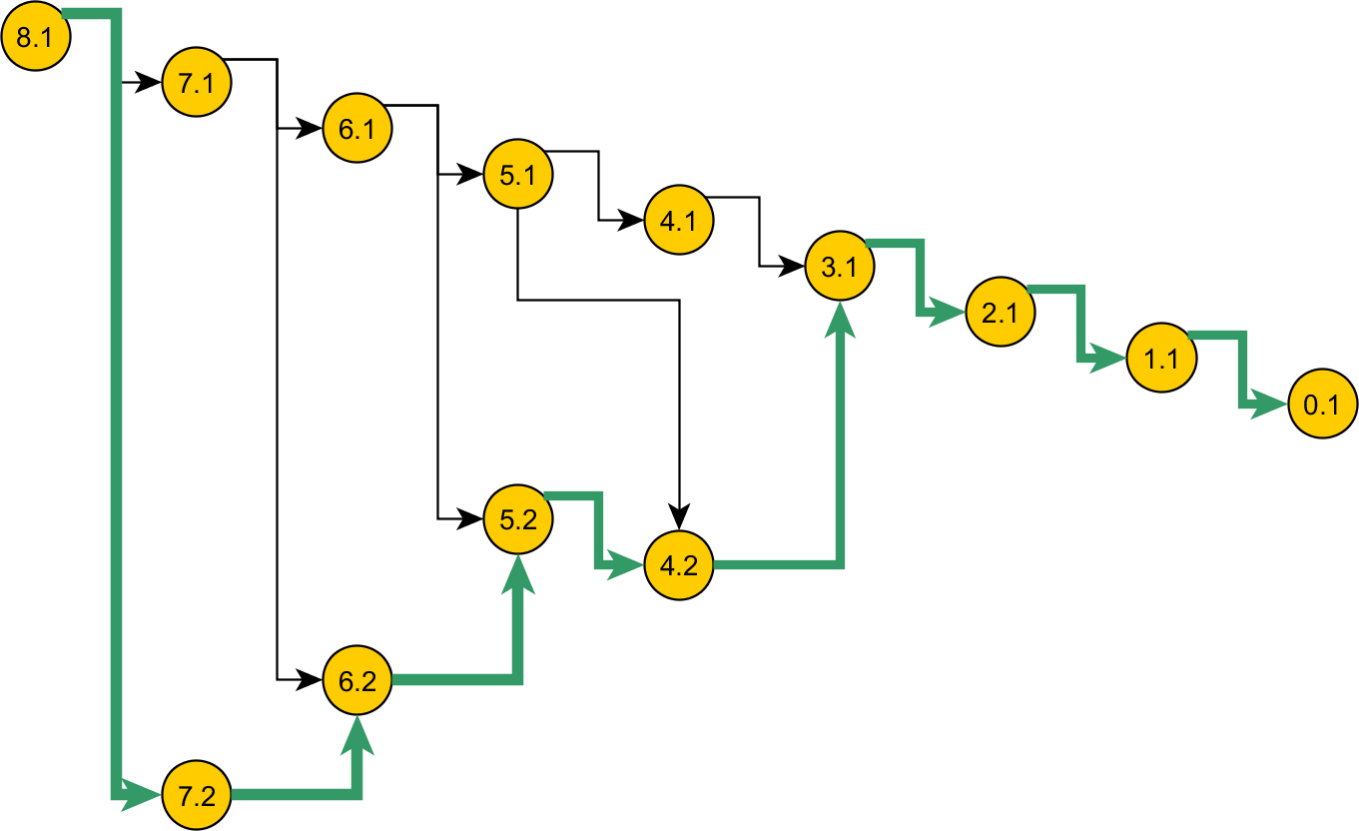
Space of genealogies as domain of sampling. The paths in the diagram constitute the space of genealogies for data in Table 1. Total number of genealogies grows exponentially with the sum of the number of alleles and the mutation count present in data. A sample path from this space is marked. Nodes represent the ancestral configurations (*AC*) and their labels have format: [*eventstoMRCA*] :[*serial-no.*]. The *AC*s are grouped by their *eventstoMRCA* which acts as a sequential step in the sampling. A particular branch is selected with chance proportional to the weight specified in the proposal.

*GProposal* implements SIS via *Proposal_ w_Prob*. It recursively builds the sample and its probability, at each step using the alternatives in Eq. (1) (The previous version of our framework already calculated the exact probabilities, reducing programming effort). Figure 5 illustrates SIS in a coalescent process. *GProposal* specifies the SIS of all coalescent models based on Eq. (1). Specific proposals (e.g., Eqs. (6), (7) and (8)) need only implement the abstract method *proposalWeight*. The program design suits Monte Carlo well, because it localizes errors and limits the scope of problems associated with a specific proposal. It also stabilizes results when comparing sequential sampling schemes: general optimizations might improve the efficiency of several schemes, but the implementation of each scheme shares the gain, thereby maintaining their relative efficiencies.

### The infinite-sites model K69

**Figure 6:**
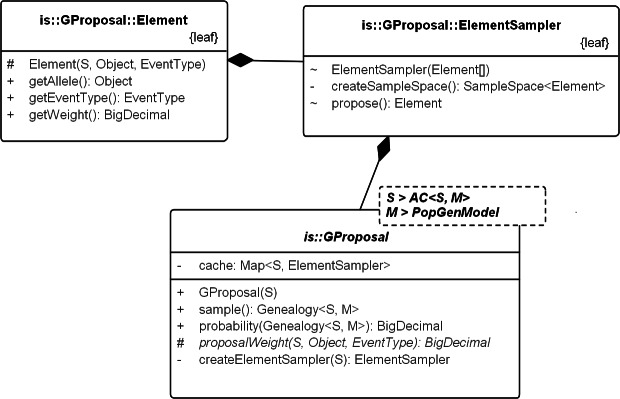
UML diagram explaining the internals of GProposal. GProposal implements *Proposal_w_Prob* with *Genealogy* for type parameter *X*. It has two central methods: (1) *sample()* and (2) *probability(genealogy)* whose implementations are correlated (second method reuses parts of the first) to be efficient. Branch weights are encapsulated in structure *Element* and one *Element* is proposed by the *ElementSampler* at each sequential step of the sampling. As such, new proposals need only proivde the specification of the proposal equation via *proposalWeight(.)* method. All the standard proposals have been implemented in this way. The implementation of SD proposal is shown in Fig. 7.

**Figure 7:**
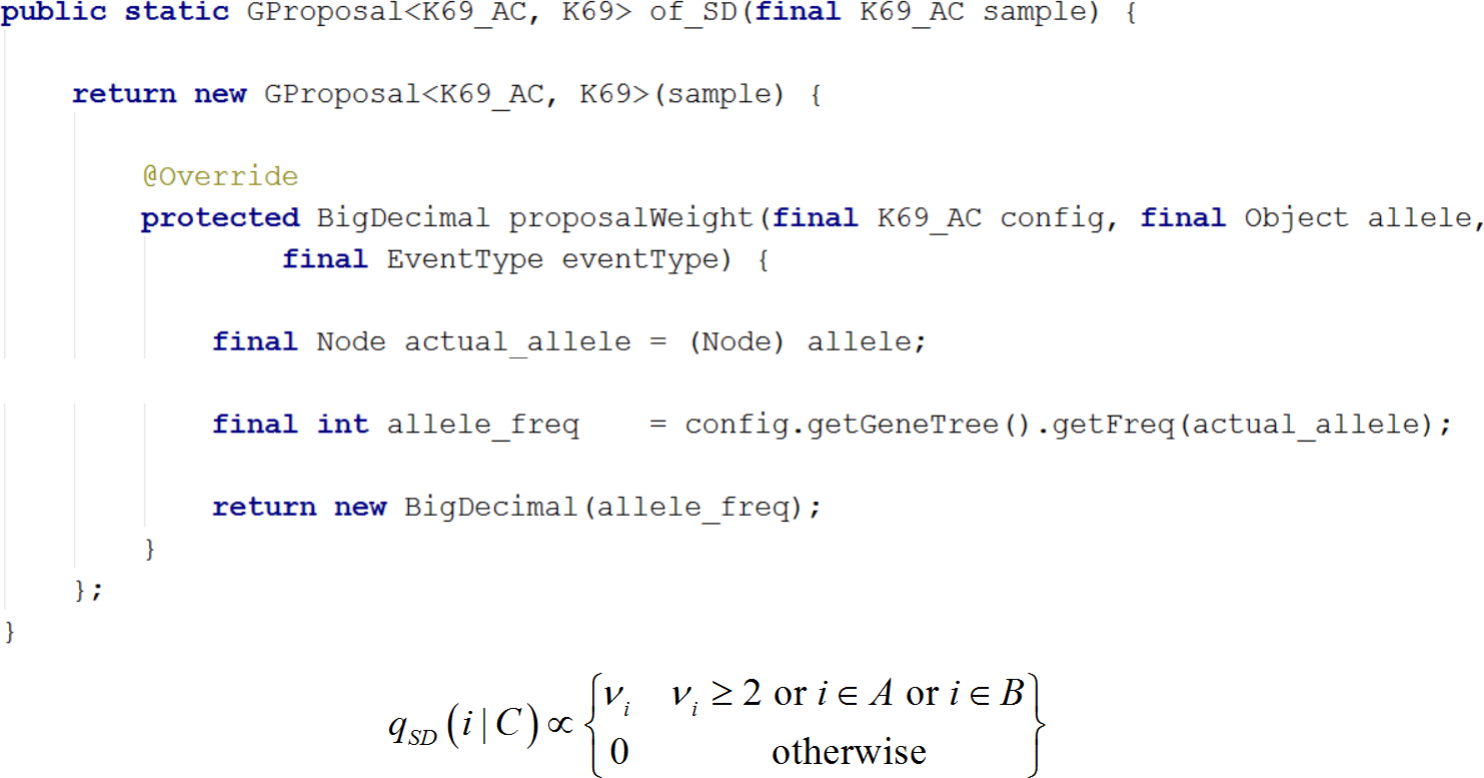
Writing a new proposal. Demonstrates writing of a new proposal using the SD proposal. Note that the implementation is a close translation of the corresponding equation.

For the infinite-sites model of mutation (*K69*), we implemented three standard proposals (Eqs. (6), (7) and (8)) with the abstract method *proposalWeight*, as described above. If the root (i.e., the state of the MRCA) is unknown, the user has two choices: (1) repeat the computation by hand for all possible roots (future versions will automate the repetition); or (2) follow the accepted expedient of substituting the consensus sequence for the root McMorris, 1977. The proposals are collected in *GProposals_K69* (see Fig. 6), which follows the factory design pattern (Gamma et al., 1995). The framework for exact probabilities already specifies the interface *K69_AC* of the ancestral configuration under infinite-sites model of mutation, so we used it to specify the three proposals. Figure 7 illustrates the implementation of SD Proposal, which demonstrates that proposals can be written compactly from the corresponding equations, leaving the framework to encapsulate the details.

### Multiple parameters

Most population genetic models with multiple parameters (including model K69) lack analytical expressions for the corresponding likelihoods, so computers are essential for calculating maximum likelihood estimates (MLEs). Separate likelihood computations for each set of parameter values is often inefficient, because some models (e.g., K69) permit computations for each set to reuse every realization, vastly improving performance. Different proposals exploit this opportunity differently, however. SD, e.g., does not use the parameter value corresponding to mutation, EGT uses scaling (Eq. 12 in Griffiths & Tavare, 1994), and HUW indirectly avoids the parameter value by using asymptotics from Eqs. 12 and 13 of Hobolth, Uyenoyama & Wiuf (2008). Computations for different proposals reuse realizations differently, so a poor design might easily require a programmer to specify a proposal twice. In contrast, our framework avoids duplication of effort and allows a single proposal specification with a single set of parameter values to sweep through several sets of parameter values by specifying a separate implementation for reusing realizations.

### Parallel proposals

The framework design is object-oriented, thereby promoting the natural use of multiple computer cores. Generally, it emphasizes modularity over optimization of running times, enhancing platform-independence and the possibility of running the framework on common multi-core machines. Multiple schemes can run on separate *threads*, reducing computing time and providing direct visual feedback on their running times. Some proposals both increase statistical power and reduce computing time relative to other proposals (e.g., SD over EGT) but more typically, a nuanced trade-off occurs.

### Tests and coverage

The expansion of the framework over time must not break existing features and design contracts (Freeman & Pryce, 2009). Thus, extensibility puts constraints on, e.g., the graphical user interface. *Automated unit tests* can permit debugging to remain manageable and coverage of *checked exceptions* (known causes of disruption) to expand. The *coverage* (the proportion of code lines that the unit tests execute) is an accepted figure of merit for unit tests. Table 2 provides the number of tests and coverage for the packages in the framework. Typically, more than 70% coverage should inspire confidence (Limjap, 2008).

**Table 2:** Tests and coverage for the framework. The table shows the number of tests and the percentage of coverage for various packages in the framework. Typically, 70% coverage is considered stable.

Test area	Number of tests	Coverage (line)
Common	47	70%
Model	10	75%
Data	31	92%
Phylogeny	11	86%
Recursion	23	62%
Statistic	27	84%
Providers	30	61%
*Importance sampling*	*62*	76%
	*241* (total)	*75.75%* (avg.)

### Features

Some features of the framework follow, along with some notes on each feature. Readers can consult the project website for more information. Framework version numbers are indicated in square brackets, and Version *1.4.2* retains all features in version *1.3.0*. Version *1.4.2* corresponds to this article; Version *1.3.0*, to the article Tewari & Spouge (2012).

1. [*1.3.0*] *Support for two models: Infinite Alleles Model (*KC64*) and Infinite Sites Model (*K69*)*. The models include mutation under the standard neutral coalescent model of a single, well-mixed population of constant size over time. Data for *K69* are read from an *xml* file; data for *KC64*, from a string literal within the framework.
2. [*1.3.0*] *Checks Phylogeny of binary data using Gusfieldś algorithm or the Four Gamete‘s algorithm. The user chooses the algorithm.*
3. [*1.3.0*] *Draws Phylogeny of *K69* data.* The framework uses Gusfield‘s algorithm to build the gene tree.
4. [*1.3.0*] *Exact computation of various model statistics.* For *K69* and *KC64* on small datasets, the framework can: (1) compute the exact likelihood and the probabilities of ancestral configurations; (2) count and build ancestral configurations and genealogies; (3) profile the recursion cache, which can be useful in improving exact algorithms.
5. [*1.4.2*] *Maximum Likelihood Estimation (MLE) using exact computation.* The MLE can be computed for *K69* and *KC64* on small datasets. The user specifies the minimum, maximum, and the increment for values of the population mutation rate.
6. [*1.4.2*] *Smart Data Integration.* Given a job, the *Data Panel* automatically lists all relevant datasets in (*xml*) files or string literals. The user can easily open and edit *xml* files within the application, which updates jobs as it auto-detects files added or deleted from the underlying system. The framework can also interpret data intended for the Infinite Sites Model *K69* for use with the Infinite Alleles Model *KC64*.
7. [*1.4.2*] *Maximum Likelihood Estimation (MLE) using Importance Sampling(IS).* Equation (10) below defines the effective sample size. For *K69* on datasets of moderate size, the framework can compute likelihoods for any combination of available proposals, including EGT, SD, and HUW. In addition, a programmer can simply specify a new proposal to add it to the framework. A user can run multiple proposals simultaneously to measure their relative efficiencies and limit the sampling by realizations (to estimate effective sample sizes) or by time (to estimate effective sample sizes per running time), with results available in either textual or graphical formats. To benchmark proposals, the framework can plot its likelihood estimates next to exact likelihoods (either provided or computed); during execution, it can also track and report various measures (e.g., standard errors, effective sample sizes, etc.).
8. [*1.4.2*] *Benchmarking Performance of Competing Proposals.* The framework simulate datasets to compare measures associated with different proposals over a grid of parameters like mutation rates and alleles in the gene tree. As above, the user can choose to present key metrics as graphs or texts.

## Results & Discussion

**Figure 8:**
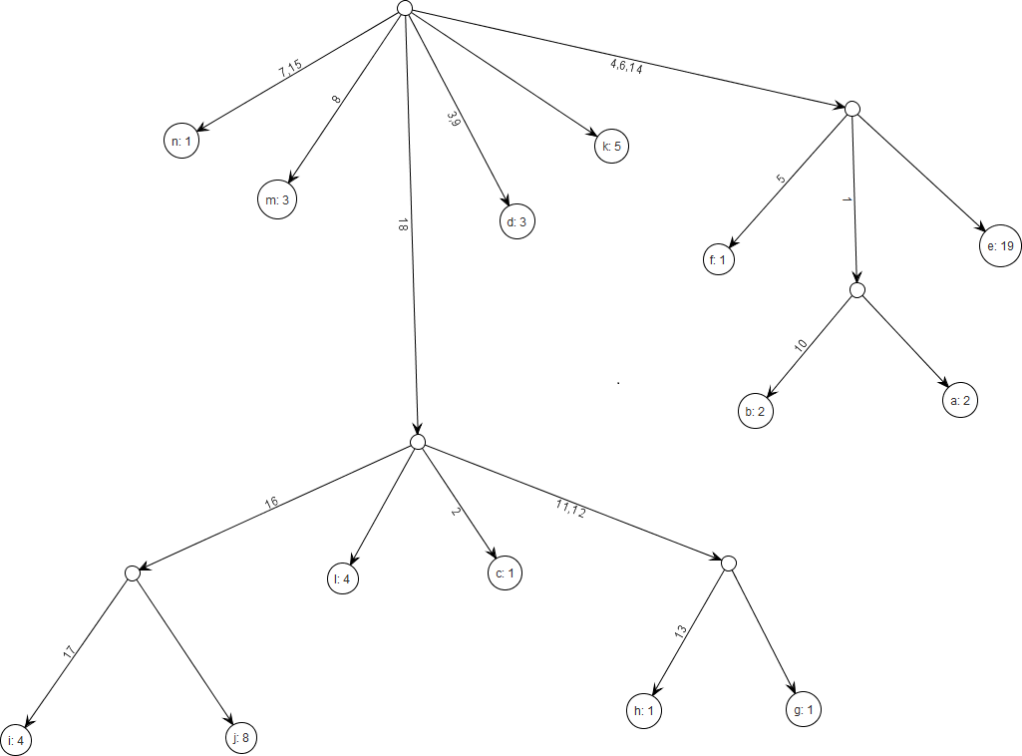
Gene tree for the benchmark data set in Griffiths & Tavare (1994). There are 18 segregating sites and 14 distinct alleles and the total sample size is 55. The figure was drawn using the framework.

Our figures and results generally follow the presentation of Hobolth, Uyenoyama & Wiuf (2008). Figure 8 displays the gene tree for the dataset of Griffiths & Tavare (1994), a standard benchmark for comparing proposals. To compare the EGT, SD, and HUW proposals, we used effective sample size (ESS),

(10) \begin{eqnarray*} E S S=\frac{R}{1+{\mathrm{var}}_{q}\left(w\left(Y\right)\right)}, \end{eqnarray*}

where *R* is the number of realizations (samples), and *w* is the corresponding importance weight (Liu, 2002, p.35). Loosely, ESS quantifies the similarity of the target distribution to the trial distribution, so SIS improves as the ESS increases.

### Validating MLE and the maximum likelihood

The framework computed the likelihood curve (Table 3). Table 4 for the MLEs resulting from each proposal is visually consistent with figures in Wu (2009) and verifies results for both standard error and ESS in Hobolth, Uyenoyama & Wiuf (2008), namely, that the SIS performance order is EGT < SD < HUW. The running times for SD and EGT were nearly equal, however, and about half of the running time for HUW. Within the framework, all proposals share the same runtime infrastructure, so the accuracy in HUW comes at the price of approximately doubling the running time per realization.

**Table 3:** Validating MLE of the mutation rate using multiple proposals. MLE is computed in the range [1.0, 10.0] with an increment of 0.1 using multiple proposals; corresponding published values are included from Wu (2009).

Proposals	MLE		Sample size	
	Published	Framework	Published	Framework
EGT	4.8	4.8	200,000	100,000
SD	[4.5, 5.0]	4.9	100,000	100,000
HUW	[4.5, 5.0]	4.9	100,000	100,000

**Table 4:** Estimating likelihood at the MLE of mutation rate 4.8 by multiple proposals for fixed number of realizations (100,000). Published values are compared against the values computed using the framework. Exact likelihood is 8.71E–20 (Wu, 2009).

Proposals	Published	Framework			
	Likelihood	Likelihood	Std. error	ESS	Time(s)
EGT	7.76E–20	7.57E–20	8.31E–21	82	1347
SD	9.33E–20	9.14E–20	5.41E–21	283	1046
HUW	8.70E–20	9.01E–20	3.75E–21	572	2160

### Confirming proposal order

**Figure 9:**
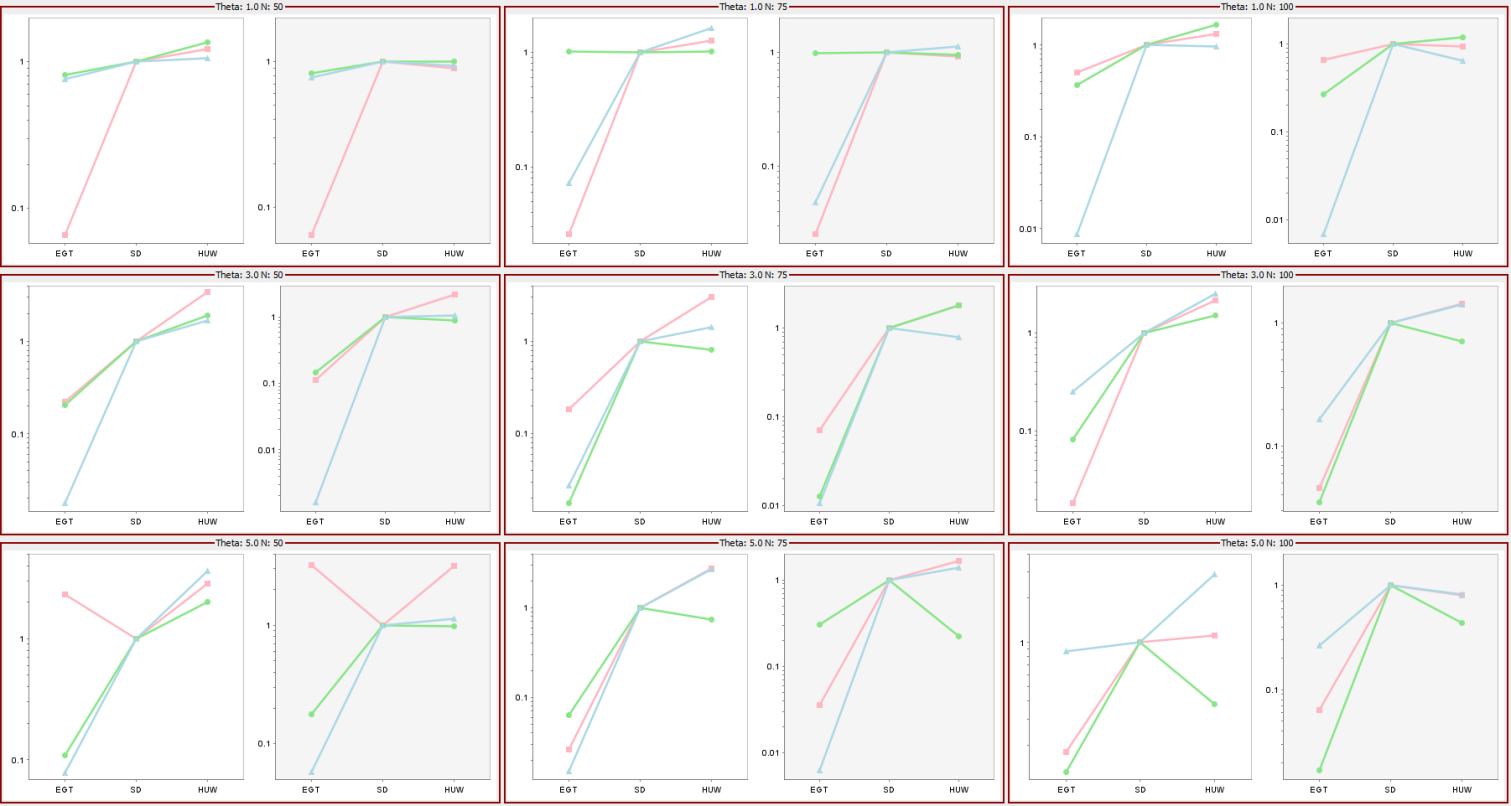
Significance of time in proposal efficiency. Three data sets are simulated under each combination of mutation rates (=1, 3, 5) and sample sizes (*n* = 50, 75, 100). Scaled ESS values (to the SD proposal) are estimated for each data set. All values are scaled to the ESS of the SD proposal, which corresponds uniformly to 1. Each cell has two plots: left panel verifies the proposal order for fixed number of observations but the right panel shows that when the running time is fixed, the increase in ESS may not be always decisive (e.g., HUW and SD).

Hobolth, Uyenoyama & Wiuf (2008) investigated the performance of the three proposals by comparing ESSs as mutation rates and data sample size varied, their Fig. 6 showing that the ESSs for three proposals have a stable relationship, EGT < SD < HUW. Using the same mutation rates and data sample sizes, our simulations independently confirmed their results as follows (Fig. 9). For each cell, the tool *ms* (Hudson, 2002) or *msms* acting as a cross-platform fallback (Ewing & Hermisson, 2010) simulated three independent sets of samples (denoted by different colours in the figure) for the corresponding mutation rate and data sample size. Within each cell, there are two plots. The left-hand panels show results if we limited all simulations to 108,000 realizations (approximating the 100,000 used in Fig. 6 of Hobolth, Uyenoyama & Wiuf (2008)); the right-hand panels, if we limited all samplers by time, terminating them when the slowest one completed 108,000 realizations. The left panels of each cell verify the results in Fig. 6 in Hobolth, Uyenoyama & Wiuf (2008).

### Effect of running time

Although the left panels of each cell verify the results in Fig. 6 in Hobolth, Uyenoyama & Wiuf (2008), the right panels show that when the figure of merit is *ESS per running time*, SD improves relative to HUW, but EGT does not. Thus, although HUW requires more time than EGT to produce a realization, the improvement in the ESS more than compensates. HUW also requires more time than SD to produce a realization, but here the improvement in the ESS does not compensate so decisively. For the real dataset in Fig. 8, Table 5 tells the similar story. The message is clear: substantial improvements in the ESS for a fixed number of realizations do not always translate into a substantial practical reductions in the errors of estimates.

**Table 5:** Estimating likelihood at the MLE of mutation rate 4.8 by multiple proposals under fixed time (3000 s). Proposals EGT and SD both sample more observations than HUW when run under the fixed time. The conclusions from Table 4 do not change. But, note that though EGT still ranks the same, the improvement of HUW over SD is less dramatic due to the difference in the number of realizations. Thus, running time should be included in comparing proposals.

Proposals	Likelihood	Std. Error	ESS	Realizations
EGT	8.43E–20	1.14E–20	54	212,277
SD	9.21E–20	5.66E–21	264	280,740
HUW	9.54E–20	5.16E–21	340	121,855

### New mixed proposal via exact samplers

Wu (2009) motivated expanding the size limits on exact analysis of coalescent data by pointing out that exact analysis can validate Monte Carlo estimates. In the same spirit, we developed a framework for exact recursive computation of coalescent probabilities (Tewari & Spouge, 2012). Our present framework is a seamless extension of our recursive framework, and it permitted us to implement a new proposal, as follows.

**Figure 10:**
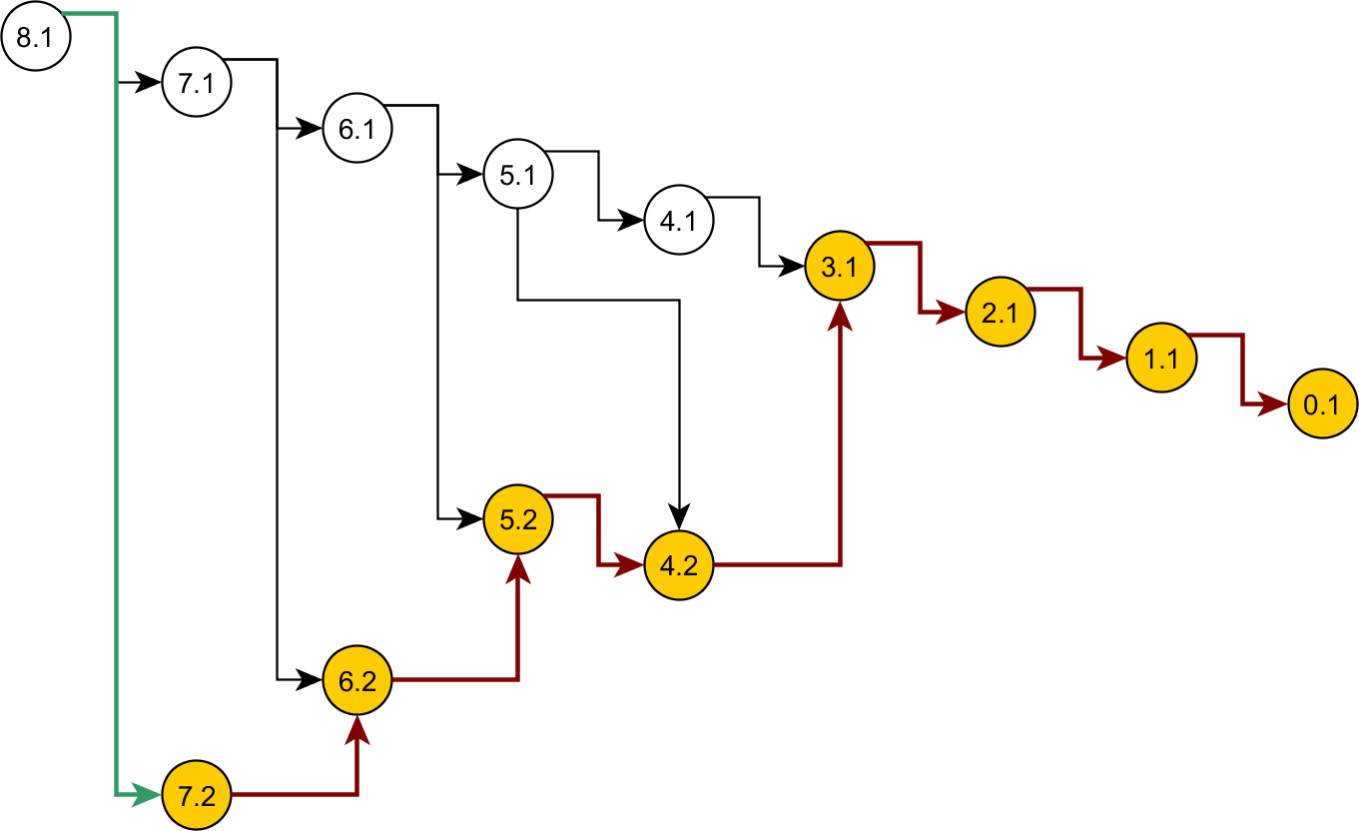
Genealogy of the exact singleton sampling for data in Table 1. Genealogy of the mixed-exact proposal contains a sub-graph (see text). Sub-graph nodes are highlighted, red arrows indicate exact sampling, and the green arrow indicates sampling via the delegate proposal.

Some nodes of the full ancestral graph permit exact sampling of transitions away from them. On the corresponding subgraphs, the framework can compute the most efficient proposal possible (the target distribution itself), while using a *delegate proposal* elsewhere. We call this idea *Exact Subgraph Sampling*. Consider, e.g., “singleton nodes”, nodes of the ancestral subgraph whose sequences all have multiplicities *ν_i_* = 1, leading to an Exact Subgraph Sampling scheme, we call *Exact Singleton Sampling*. Figure 10 highlights a sampling path using exact singleton sampling for data in Table 1. For the real data in Fig. 8, the exact distribution for the singleton nodes can be computed under 10 s with average memory, whereas the full graph takes more than a day on a high-end PC (Wu, 2009). Because they are beyond the purview of this article, detailed results for Exact Singleton Sampling will be presented elsewhere, but preliminary results for the data in Fig. 8 suggest that Exact Singleton Sampling improves the ESS per computational time for HUW by a factor of about 4.

## Conclusions

Running time is a significant practical consideration when comparing the computational efficiency of different importance sampling proposals. With ESS per running-time replacing ESS as a figure of merit used in Hobolth, Uyenoyama & Wiuf (2008), the Hobolth-Uyenoyama-Wiuf proposal (HUW) retains only a small edge over the Stevens-Donnelly proposal (SD). In this case, therefore, the expected sample size (ESS) per running time and the ESS agree HUW as the optimal proposal, but disagree quantitively on the relative improvement. Running time is an important practical consideration, however, so when comparing proposals on a level playing field within a single computational framework, the ESS per running time should receive more weight than the ESS as a figure of merit. Thus, evaluations within computational frameworks like ours should be preferred to *ad hoc* evaluations of importance sampling schemes (ISSs). As an aside, our framework includes exact computations (Tewari & Spouge, 2012), so it easily implements the new proposal, Exact Singleton Sampling, described above.

Our project website (http://coalescent.sourceforge.net) contains open source code in the Java programming language under the GPLv3 license. Following the spirit of open science (Stodden, 2013a; Stodden, 2013b; Stodden, 2013c), our software reflects a systems approach. For instance, our claims can be verified by running our software, which comes with several test cases backed by a large test coverage. The software itself is scalable and its user interface is intuitive, requiring only a basic familiarity with the theory, and our Supplemental Information contain an illustrated stepwise instruction manual. The present framework augments our earlier framework for exact algorithms (Tewari & Spouge, 2012), also with a systems approach, adding another tool to the likelihood analysis for population genetics data under the infinite sites model of mutation.

## Supplemental Information

10.7717/peerj.1203/supp-1 Supplemental Information Peer Review Manual for using the Software Manual for using the Software for verifying the claims in the manuscript.

## Additional Information and Declarations

### Data Availability

The following information was supplied regarding the availability of data: http://coalescent.sourceforge.net/.
