# Supplementary material for: Coalescent: an open-science framework for importance sampling in coalescent theory
Source: PeerJ. 2015 Aug 18;3:e1203. doi: 10.7717/peerj.1203 (PMC4548476; doi:10.7717/peerj.1203)
Supplement: Supplemental Information — Manual for using the Software for verifying the claims in the manuscript. [file peerj-03-1203-s001.pdf]

Peer-Review Manual: Using coalescent to verify claims in the Manuscript

See companion video at: http://youtu.be/phNY54AvGfU.

See ERRATA *at the end of this document.*

System Requirements

`coalescent` is supported only on 64 bit platforms (Windows, Mac-OS-X, Linux) as it involves intensive computation. While 64 bit platforms allow larger memory space, they also consume twice as much memory (compared to 32 bit systems), simply to store a larger address space. This is a well known trade-off.

Jobs would require different amounts of memory depending on various factors but 4 GB of dedicated memory to the underlying Java Virtual Machine (JVM) process would be sufficient for running all the jobs. The dedicated memory to the JVM is allocated during program launch and is typically set to 1/4th of the system`s free memory. If your system does not have enough free memory, the program may become unresponsive for computationally demanding jobs. If the system has enough free memory (say, 8GB) but that would only allocate around 2GB of dedicated memory, you can allocate the required memory manually. Go to `INSTALL-DIRECTORY/etc/coalescent.conf` and edit the following:

change the line

```
default_options="--branding coalescent -J-Xms24m"
```

to

```
default_options="--branding coalescent -J-Xms24m –J-Xmx4000m"
```

Quick Tour of coalescent via Screenshots

Below is a screenshot of the whole interface under Windows7. Mac-OSX and Linux have the looks slightly different but the content and structure will be the same. This guide will use

Windows7 for illustration but the instructions apply to all three (Windows, Mac-OSX and Linux) as well.

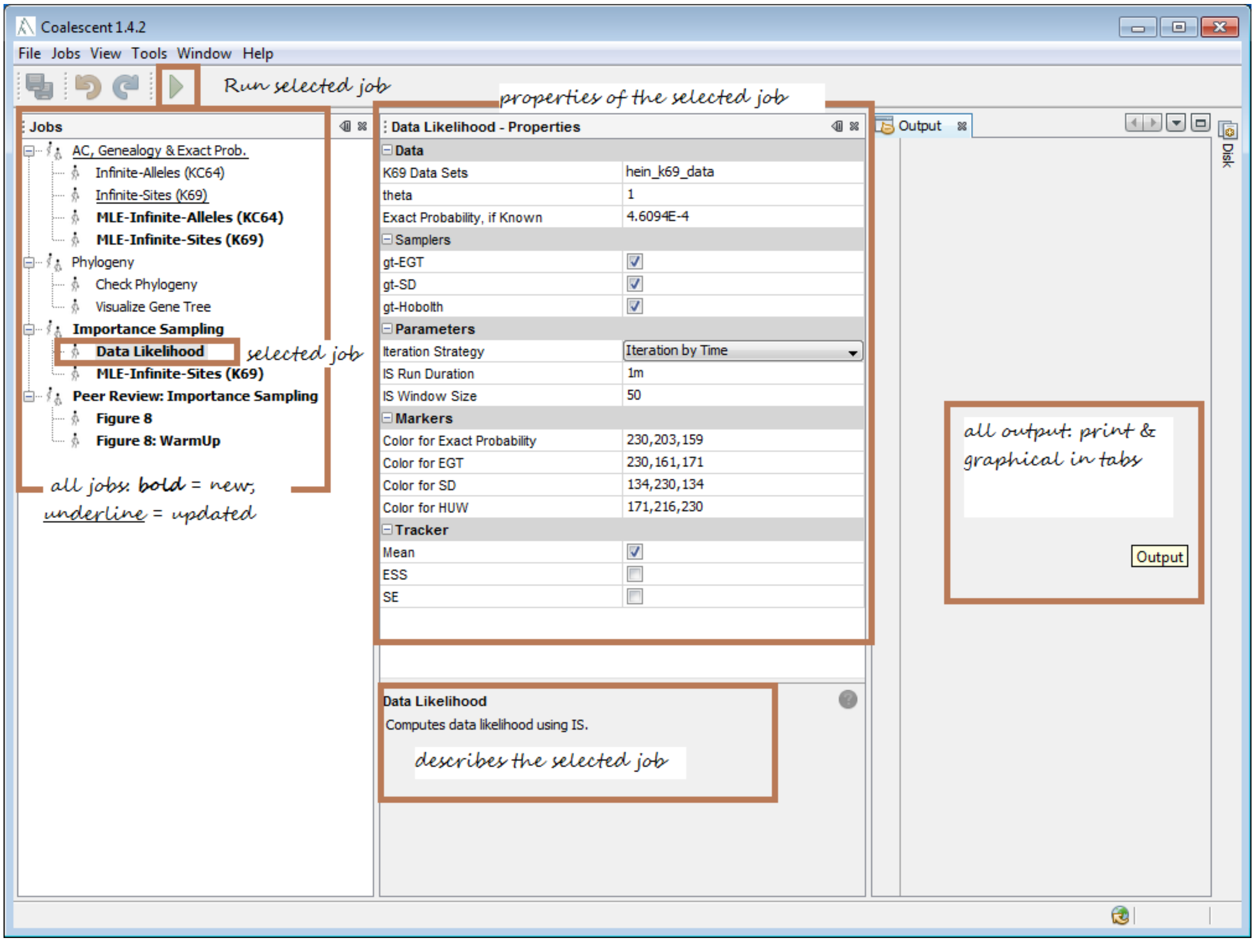

Figure 1 Jobs under Review. See errata for changes in job names.

The jobs under **Importance Sampling** and **PeerReview:Importance Sampling** are only under scrutiny for this manuscript. These jobs can be used to verify all the claims in the manuscript. We will describe these jobs in detail and show how to reproduce the results.

Jobs

The job **Data Likelihood** computes likelihood of data under the infinite sites model. The job **MLE-Infinite-Sites (K69)** computes MLE of the mutation parameter for data under the infinite sites model. Note that these two jobs are similar except for the range of mutation parameters required in the latter.

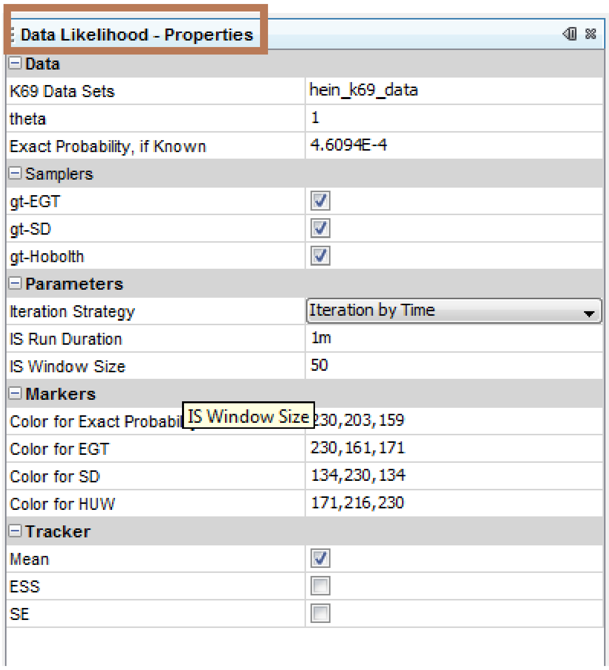

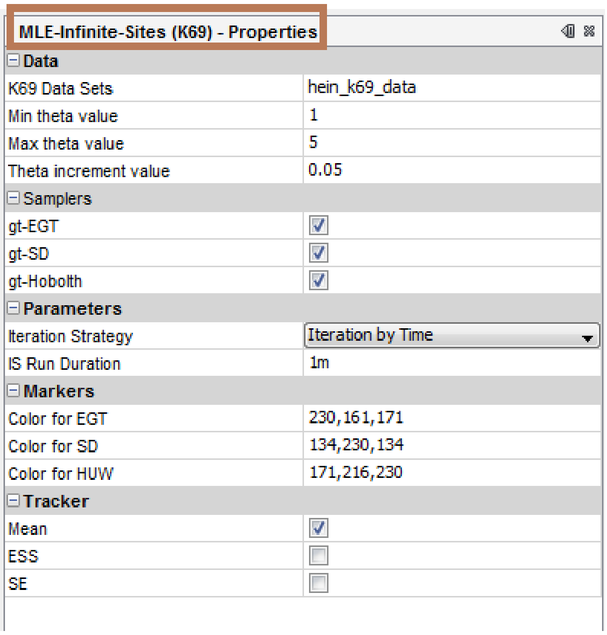

The job **Figure 9** computes Figure 9 in the manuscript corresponding to the benchmark of Figure 6 in Hobolth, A., Uyenoyamay, M. K., & Wiuf, C. (2008). The job **Figure 9: WarmUp** is exactly similar to job Figure 9 except the the number of realizations of the importance sampling is 100 times more in the latter. This was to keep this simulation study comparable to the benchmark. However, this takes long time and to give the user a quick overview, we have created the job 'Figure 9: WarmUp'. It finishes in minutes with a clear view of the progress in each cell.

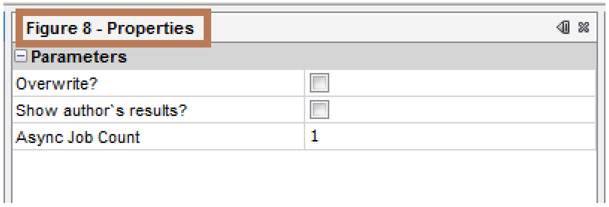

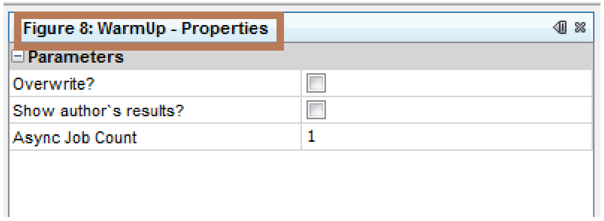

Claim 1: Table 3 – Computing MLE using Multiple Proposals

The job **MLE-Infinite-Sites (K69)** can be used to verify this table. Select '`K69 Data Set`' to be `gt94_k69_data`. Set '`Min theta value = 1.0`', '`Max theta value = 10.0`', '`Theta increment value = 0.1`'. Make sure all the samplers, `gt-EGT, gt-SD, gt-HUW` are checked. Under Parameters choose the iteration strategy i.e., how the number of realizations of the importance sampling is counted. There are three options: `by-OrderSize-Unit`, `by-Time` and `by-SampleSize`. Choose `by-SampleSize` and set

'IS Run Duration = 100000'. Run the job and inspect the textual and graphical output to verify data in Table 3 of the manuscript.

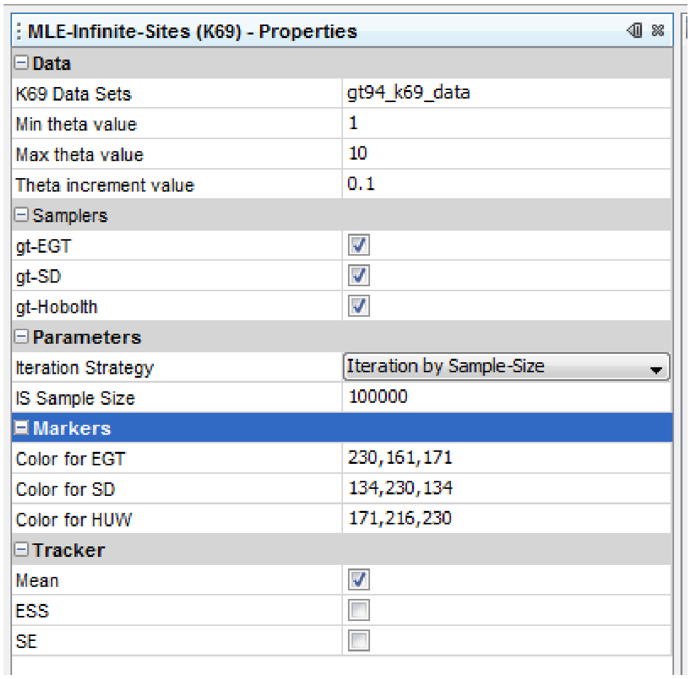

Claim 2: Table 4 – Estimating Likelihood at MLE by Multiple Proposals

The job **Data Likelihood** can be used to verify this table. Select 'K69 Data Set' to be gt94_k69_data. Set 'theta = 4.8', and 'Exact probability = 8.71E-20'. Make sure all the samplers, gt-EGT, gt-SD, gt-HUW are checked. Under Parameters choose the iteration strategy i.e., how the number of realizations of the importance sampling is counted. There are three options: by-OrderSize-Unit, by-Time and by-SampleSize. Choose by-SampleSize and set 'IS Run Duration = 100000'. Run the job and inspect the textual and graphical output to verify data in Table 4 of the manuscript.

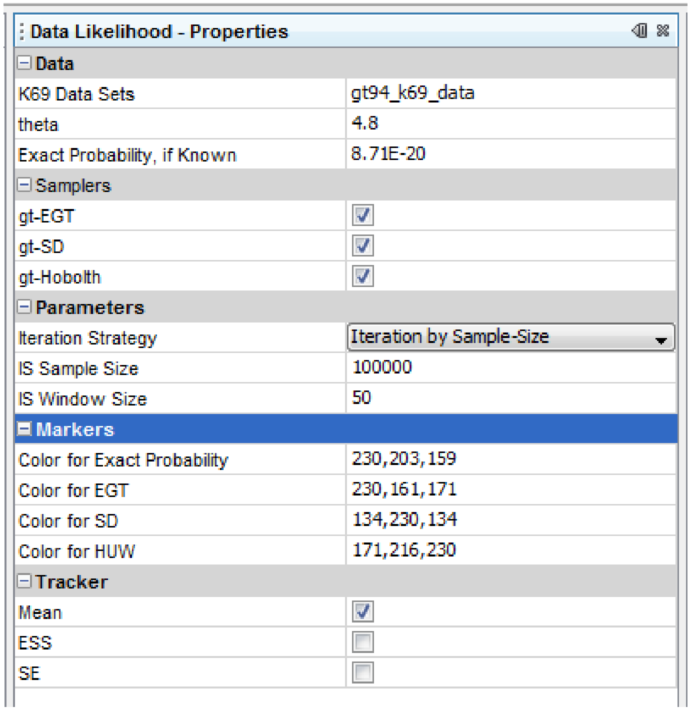

Claim 3: Figure 9 – Simulation Results showing Significance of Time in Proposal Efficiency

The job **Figure 9** can be used to verify this figure. To display the author`s results check ‘Show author`s results?’ and run the job. It immediately displays Figure 9 with associated data. To compute the results afresh, check ‘Overwrite?’ and run the job. Note that the author`s data are not lost by this and can be displayed again. The label ‘overwrite’ means overwriting any previous user computation; if unchecked, starts the computation where it was left off (the application persists the state of computation because this is a long running job) either by cancelling the job or an application exit. If job had finished before, running the job would immediately display the results. The property ‘Async job count’ lets run multiple cells in parallel. This property appears only if the underlying system has enough number of cores to make parallel execution benefinicial.

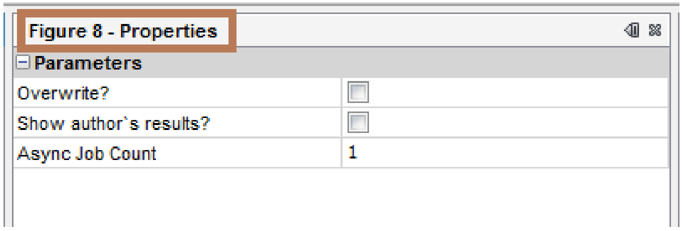

Errata

1. Figure 1 has job names with labels “Figure 8” and “Figure 8: Warm Up”. They should instead be “Figure 9” and “Figure 9: WarmUp”.

References

Hobolth, A., Uyenoyamay, M. K., & Wiuf, C. (2008). Importance Sampling for the Infinite Sites Model. *Statistical Applications in Genetics and Molecular Biology, 7*(1).
